# Efficacy and safety of transradial versus transfemoral approach for secondary access in transcatheter aortic valve implantation: a systematic review and meta-analysis

**DOI:** 10.1097/MS9.0000000000003562

**Published:** 2025-07-14

**Authors:** Zaryab Bacha, Noor Fatima, Izhar Muhammad Shah, Muhammad Haris, Muhammad Hamad Ali, Naveed Ahmed Khan, Shahzaib Khan, Muhammad Qaiser Shah, Ayiz Jan, Sana Ullah Khan, Umar Tariq, Iram Raza, Mohammad Ebad Ur Rehman, Muhammad Sheraz Hameed, Syed Rafay Hussain Zaidi, Rahmat Gul Omarzai

**Affiliations:** a Department of Medicine, Khyber Medical College, Peshawar, Pakistan; b Department of Medicine, Gomal Medical College, Dera Ismail Khan, Pakistan; c Department of Medicine, Khyber Teaching Hospital, Peshawar, Pakistan; d Department of Medicine, Lady Reading Hospital, Peshawar, Pakistan; e Department of Medicine, Saidu Group of Teaching Hospitals, Swat, Pakistan; f Department of Medicine, Federal Medical and Dental College, Islamabad, Pakistan; g Department of Medicine, Rawalpindi Medical University, Rawalpindi, Pakistan; h Department of Medicine, Parkview Medical Center, Pubelo, United States America; i Department of Medicine, Nangarhar Medical University, Jalalbad, Afghanistan

**Keywords:** aortic stenosis, femoral artery, radial artery, transcatheter aortic valve implantation

## Abstract

**Background:**

Transcatheter aortic valve implantation (TAVI) requires a primary access to deliver the valve and a secondary access for angiographic guidance. Although transfemoral access (TFA) is most commonly employed, alternative access sites are gaining traction. This systematic review and meta-analysis compares the efficacy and safety of transradial (TRA) and TF secondary access.

**Methods:**

A literature search was performed on MEDLINE, Embase, Cochrane, and Clinicaltrials.gov from their inception to January 2025. Risk ratios (RR) with 95% confidence intervals (CI) were pooled using the Mantel–Haenzel method for dichotomous outcomes. Mean differences (MD) with 95% confidence intervals (CI) were pooled using the inverse variance method for continuous outcomes. Random-effects meta-analyses were undertaken.

**Results:**

Seven studies, with 15 283 patients, were included. TRA had a significantly lower risk of vascular complications (RR 0.47, 95% CI 0.25–0.89), major vascular complications (RR 0.45, 95% CI:0.27–0.73), bleeding (RR 0.53, 95% CI 0.36–0.78), major bleeding (RR 0.55, 95% CI 0.34–0.90), stroke (RR 0.62, 95% CI 0.39–0.99), all-cause mortality (RR 0.45, 95% CI 0.36–0.57), and acute kidney injury (RR 0.48, 95% CI 0.38–0.60). Both groups were comparable in terms of life-threatening bleeding, myocardial infarction, permanent pacemaker requirement, length of hospital stay, contrast volume, procedure time, and fluoroscopy time.

**Conclusion:**

TRA is associated with superior outcomes compared to TFA for secondary access in TAVI. Furthermore, large-scale randomized trials are needed to clarify the most optimal access sites for TAVI.

## Introduction

Transcatheter aortic valve implantation (TAVI) involves inserting a bioprosthetic valve through a catheter and placing it within the existing diseased aortic valve^[1]^. This procedure represents a significant advancement in the treatment of severe aortic stenosis (AS), offering a viable alternative for the patients who are deemed unsuitable or at high risk for conventional surgical aortic valve replacement (SAVR) over the last decade.^[2]^ Transfemoral artery access (TFA) is the preferred method for the primary access site in TAVI due to its lower complication rates compared to other access points. The use of TFA has significantly increased, from 57.1% since the inception of TAVI to 95.3% in 2019. In 2019, femoral access was utilized in 93.65% of high-risk patients, 96.2% of intermediate-risk patients, and 97.8% of low-risk patients^[3]^. In addition to the main large-bore access used for delivering transcatheter heart valves, a secondary access point is utilized for pigtail placement and aortic root angiography to assist in guiding the implantation. This secondary access is most commonly achieved through a second TFA puncture, typically through the contralateral femoral artery during transfemoral-TAVR^[4]^. However, approximately one-quarter of vascular access site complications, including one-third of major vascular complications in TAVR, are associated with the transfemoral secondary access^[5]^.

In recent years, transradial access (TRA) has become the preferred method for most coronary procedures due to its advantages over TFA in terms of faster post-procedural recovery, greater patient preference, reduced vascular access site complications, bleeding, and mortality^[6]^. While root aortography can be easily performed using the radial approach, peripheral angiography and intervention can be more complex due to most equipment being designed for the femoral artery. Recently, specialized equipment has been created to facilitate peripheral vascular interventions from the TRA approach^[7]^, and these techniques could potentially be applied to TAVI procedures. Despite the growing preference for TRA in coronary procedures, there has been limited research on its use as a secondary access route for TAVI.

A recent meta-analysis has evaluated the use of TRA compared to TFA as secondary access for TAVI^[8]^. The findings were highly encouraging, revealing that among TAVI patients, secondary TRA is associated with better outcomes compared to TFA. These include significantly lower rates of major bleeding, major vascular complications, all-cause mortality, and stroke. However, several new studies^[9,10]^, have been published in recent years, providing additional valuable data on the TRA approach as secondary access for TAVI. This meta-analysis integrates these recent randomized controlled trials (RCTs) to offer a more comprehensive and up-to-date evaluation. Moreover, previous evidence has not addressed certain outcomes such as acute kidney injury (AKI), the need for a permanent pacemaker, and procedure time. Therefore, we have undertaken a systematic review and meta-analysis to compare TRA vs. TFA for secondary access in patients undergoing TAVI.

## Methods

This systematic review and meta-analysis has been conducted following the Preferred Reporting Items for Systematic Review and Meta-Analysis (PRISMA) guidelines^[11]^. The study protocol was prospectively registered on PROSPERO (CRD420250654859).

### Search strategy

A search of major databases including MEDLINE, Embase, Cochrane Library was conducted from inception to January 2025, using MeSH terms and keywords for “Transcatheter Aortic Valve Replacement,” “Femoral Artery” and “Radial Artery.” Bibliographies of relevant studies and reviews were hand-searched for additional references.

### Eligibility criteria and study selection

Studies were considered eligible for inclusion if they met the following criteria: (1) population: patients undergoing TAVI, (2) intervention: TRA, (3) comparison: TFA, and (4) study design: RCTs and cohort studies.

After removing duplicates using Rayyan, two investigators independently assessed the titles and abstracts per the eligibility criteria. Subsequently, they retrieved and assessed the full texts of the selected articles to determine their final eligibility for both qualitative and quantitative analysis. A third author was consulted in the event of any disagreements.

**HIGHLIGHTS**

- Seven studies (15 283 patients) were included in this meta-analysis.
- Radial secondary access reduced vascular complications in patients undergoing transcatheter aortic valve replacement.
- Risk of bleeding, stroke, mortality, and acute kidney injury were lower with radial access.

### Data extraction

Data were extracted into a pre-piloted Excel spreadsheet, including author name, publication year, country, sample size, study design, mean age, gender, hypertension, diabetes, smoking, previous MI, dyslipidemia, primary access site, hemostasis of primary access, hemostasis of secondary access and outcomes, namely vascular complication, major vascular complication, bleeding, major bleeding, life-threatening bleeding, stroke, all-cause mortality, MI, AKI, need for permanent pacemaker, length of hospital stay, contrast volume, procedure time, and fluoroscopy time.

### Risk-of-bias assessment

To evaluate the risk of bias, the Cochrane Risk-of-Bias tool for randomized trials (RoB 2.0) was used^[12]^. This tool assesses risk of bias across five domains: (1) bias due to randomization process, (2) bias due to deviation from the intended intervention, (3) bias due to missing outcome data, (4) bias due to measurement of outcome, and (5) bias due to selection of reported results. Bias in all studies was rated as high, low, and some concerns. The included cohort studies were evaluated using the Newcastle Ottawa scale (NOS)^[13]^. Two authors evaluated bias in included studies independently. Differences in the evaluation were resolved via consultation with a third author.

### Statistical analysis

All meta-analyses were undertaken using Review Manager (RevMan) version 5.4.1. Risk ratios (RR) with 95% confidence intervals (CI) were pooled using the Mantel–Haenzel method for dichotomous outcomes. Mean differences (MD) with 95% CI were pooled using the inverse variance method for continuous outcomes. Random-effects meta-analyses were undertaken. The pooled estimates were represented as a forest plot. The Higgins *I*^2^ statistic was implemented to gauge the heterogeneity of included studies.

## Results

### Search results

**Figure 1.**
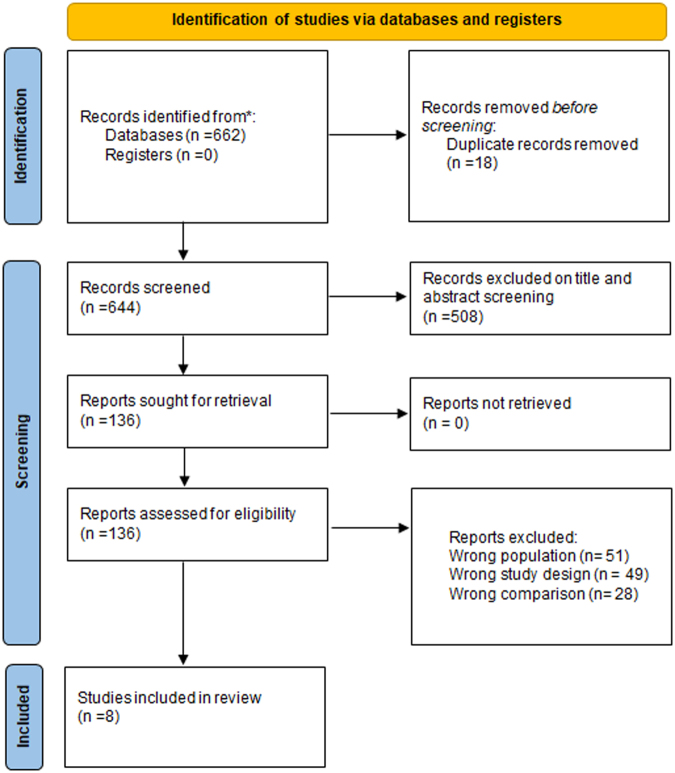
PRISMA Flowchart.

The literature search initially retrieved 662 articles. After removing 18 duplicates, 644 unique articles remained. The title and the abstract screening excluded 508 articles, leaving 136 studies for full-text screening against the eligibility criteria. Eventually, seven cohorts and one RCT was included in this meta-analysis. The selection of studies is summarized in Fig. 1.

### Study characteristics

Our meta-analysis enrolled seven cohorts and one RCT that met the predefined eligibility criteria^[4,5,9,10,14–17]^. Two studies were done in France. One study each was done in Canada, Italy, Germany, United Kingdom and the Netherlands. One study was conducted in 10 European and Canadian centers. Publication years ranged from 2014 to 2024. The total patients included were 15 462. Table 1 provides the summary of baseline characteristics for included studies.

**Table 1:** Characteristics of included studies

Study ID	Country	Study design	Sample dize	Mean Age, in years (SD)	Male (%)	HTN (%)	Diabetes (%)	Primary access site	Hemostasis of primary access site	Hemostasis of secondary access site	Follow-up
Allende 2014	Canada	Cohort	462 (127 vs 335)	79.1 (8.6)	50	87	34	Transfemoral	Surgical cutdown	Manual compression and percutaneous closure ie proglide and angio-seal for femoral access and mechaniacal compression for Radial access	30 days
Curran 2014	Italy	Cohort	87 (46 vs 41)	80.1 (7.8)	42.5	86	27.9	Transfemoral	Prostar closure device and Over the wire balloon	Over the wire balloon	30 days
Fernandez-Lopez 2019	France	Cohort	411(217 vs 194)	83	49	56	28		Proglides,Angio-seal,Protamine	Mechanical compression,Over-the-wire balloon,Covered stent	30 days
Grundmann 2024	Germany	Cohort	8851 (4505 vs 4346)	82 (6.9)	50.9	NR	27	Transfemoral (7165), Transradial (1686)	NR	NR	30 days
Jackson 2019	United Kingdom	Cohort	179 (115 vs 64)	82.0 (6.9) vs 84.9 (5.0)	54	NR	21.7	Transfemoral (179), Non-TF (20)	Balloon tamponade, balloon dilatation and aspiration thrombectomy at the primary access femoralartery following suboptimal peri-procedural anticoagulation	NR	30 days
Junquera 2020	Canada and Europe	Cohort	4949 (933 vs 4016)	8(8)	48	NR	44	Transfemoral (83.3%), transapical,transcarotid,transaortic,transsubclavian/transaxilar,transcaval (16.8%)	surgical cut-down and percutaneous closure	manual compression	30 days
Lefèvre 2019	France	Cohort	285 (76 vs 209)	84.5	44.5	NR	NR	Transfemoral	Two proglide closure devices	TR Band (Terumo Interventional Systems) or local compression	NR
Versteeg 2024	The Netherlands	RCT	238 (119 vs 119)	79.4 (6.5)	63	71	27.3	Transfemoral	vascular closure device	Angio-Seal for femoral access or TR-Band for radial access [Terumo Corporation])	30 days

### Risk-of-bias assessment

All seven cohorts were thoroughly assessed and had low risk of bias. The included RCT had some concerns of bias due to issues in the selection of reported outcomes. Risk-of-bias assessments are summarized in Supplemental Digital Content, Figure S1, available at: http://links.lww.com/MS9/A886 and Supplemental Digital Content, Table S1, available at: http://links.lww.com/MS9/A885.

### Meta-analysis of primary outcomes

#### Vascular complications

**Figure 2.**
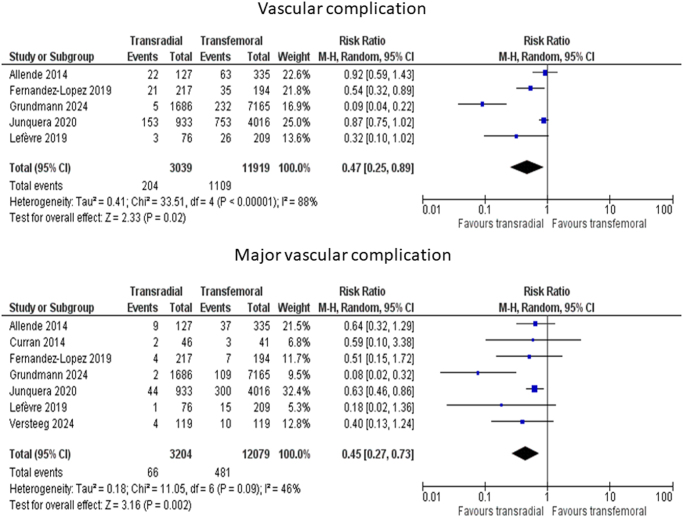
Forest plot of primary outcomes.

A total of five studies with a total sample size of 14 958 patients (TRA 3039 and TFA 11 919) assessed the vascular complications among patients in both groups. A significantly lower risk of vascular complications was observed with TRA compared to TFA (RR 0.47, 95% CI:0.25–0.89, *P*-value = 0.02, *I*^2^ = 88%). This is demonstrated in Fig. 2.

#### Major vascular complications

Seven studies with 15 283 patients (TRA 3204 and TFA 12 079) were included. TRA was associated with a significantly lower risk of major vascular complications (RR 0.45, 95% CI:0.27–0.73, *P*-value = 0.002, *I*^2^ = 46%). These results are detailed in Fig. 2.

#### Meta-analysis of secondary outcome

##### Bleeding

Four studies, analyzing 14 449 patients (TRA 2995 and TFA 11 494), reported bleeding outcomes. TRA had a significantly lower risk of bleeding compared to TFA (RR 0.53, 95% CI:0.36–0.78, *P*-value = 0.001, *I*^2^ = 63%) (Supplemental Digital Content, Figure S2, available at: http://links.lww.com/MS9/A886).

##### Major bleeding

Major bleeding was reported by five studies including 1200 patients (573 in TRA group vs. 627 in TFA group). Our analysis yielded a significantly lower incidence of major bleeding with TRA (RR 0.55, 95% CI:0.34–0.90, *P*-value = 0.02, *I*^2^ = 0%). The findings are summarized in Supplemental Digital Content, Figure S2, available at: http://links.lww.com/MS9/A886.

##### Life-threatening bleeding

Four studies with 962 patients (454 in TRA group vs. 508 in TFA group) were included. No significant difference was observed in the incidence of life-threatening bleeding between TRA and TFA (RR 0.66, 95% CI:0.32–1.34, *P*-value = 0.25, *I*^2^ = 0%) as represented in Supplemental Digital Content, Figure S2, available at: http://links.lww.com/MS9/A886.

##### Stroke

All the included studies comprising 15 462 patients (TRA 3319 vs. TFA 1143) reported the occurrence of stroke in the study population. Analysis showed that there was a significantly lower risk of stroke with TRA compared to TFA (RR 0.62, 95% CI:0.39–0.99, *P*-value = 0.04, *I*^2^ = 35%), as shown in Supplemental Digital Content, Figure S2, available at: http://links.lww.com/MS9/A886.

##### All-cause mortality

Seven studies comprising a total of 15 024 patients (TRA 3200 vs. TFA 12 024) reported all-cause mortality following TAVI. Statistical analysis demonstrated a significantly reduced all-cause mortality risk with TRA compared to TFA (RR 0.45, 95% CI: 0.36–0.57, *P*-value < 0.00001, *I*^2^ = 0%). These findings are summarized in Supplemental Digital Content, Figure S3, available at: http://links.lww.com/MS9/A886.

##### Myocardial infarction

Only three studies, including 9400 patients (TRA 1859 vs. TFA 7541) reported the occurrence of MI in patient post-TAVI. There was no significant difference between TRA and TFA (RR 0.62, 95% CI:0.25–1.51, *P*-value = 0.29, *I*^2^ = 0%) (Supplemental Digital Content, Figure S3, available at: http://links.lww.com/MS9/A886).

##### Acute kidney injury

Five studies assessed the risk of AKI in 14 536 patients (TRA 3001 vs. TFA 11 535). Findings demonstrated a significantly lower incidence of AKI with TRA (RR 0.48, 95% CI:0.38–0.60, *P*-value < 0.00001, *I*^2^ = 0%) in contrast to TFA, as depicted in Supplemental Digital Content, Figure S3, available at: http://links.lww.com/MS9/A886.

##### Permanent pacemaker requirement

Only three out of eight studies with 9500 patients (TRA 2022 vs. TFA 7478) reported the requirement of a permanent pacemaker among patient patients undergoing TAVI. Our analysis concluded no significant difference between TRA and TFA (RR 0.99, 95% CI: 0.73–1.36, *P*-value = 0.97, *I*^2^ = 55%), as depicted in Supplemental Digital Content, Figure S3, available at: http://links.lww.com/MS9/A886.

##### Length of hospital stay

Four of the included studies assessed the length of hospital stay. In a total of 9785 patients (TRA 2098 vs. TFA 7687), statistical analysis revealed no significant difference between the two approaches (MD: − 0.64 days, 95% CI: − 1.63–0.36; *P*-value = 0.21, *I*^2^ = 92%). This is represented in Supplemental Digital Content, Figure S4, available at: http://links.lww.com/MS9/A886.

##### Contrast volume

Five studies comprising 1200 patients (TRA 573 vs. TFA 627) evaluated the volume of contrast required in both approaches. Statistical analysis showed no significant variation between TRA and TFA (MD 1.23 mL, 95% CI −9.88 − 12.33, *P*-value = 0.83, *I*^2^ = 74%), as shown in Supplemental Digital Content, Figure S4, available at: http://links.lww.com/MS9/A886.

##### Procedure time

Procedure time for TAVI was reported by only three studies, comprising a total of 828 participants (TRA 451 vs. TFA 377). There was no significant difference between the two access routes (MD 6.43 min, 95% CI: −5.45 to 18.31; *P*-value = 0.29; *I*^2^ = 83%), as depicted in Supplemental Digital Content, Figure S4, available at: http://links.lww.com/MS9/A886.

##### Fluoroscopy time

Fluoroscopy time was reported by five studies, comprising 1200 patients (TRA 573 vs. TFA 627). Analysis revealed no significant difference between the two approaches for TAVI (MD 0.81 min, 95% CI −1.08 to 2.70, *P*-value = 0.40, *I*^2^ = 72%) (Supplemental Digital Content, Figure S4, available at: http://links.lww.com/MS9/A886).

## Discussion

This meta-analysis, comprised of one RCT and seven cohort studies having 15 462 participants, revealed reduced occurrence in major vascular complications, vascular complications, bleeding, major bleeding, stroke, acute kidney injury, and all-cause mortality for TRA compared to TFA, while MI, life-threatening bleeding, permanent pacemaker requirement, procedure time, contrast volume and fluoroscopy time and length of stay at hospital showed no significant difference between both groups.

TRA has emerged as an alternative to the traditional TFA approach in TAVI. Compared to TFA, TRA has been associated with a reduction in access-site complications, including vascular injuries and bleeding, which are more prevalent with the femoral approach^[18]^. While TRA is widely established in coronary interventions, its application in structural heart procedures such as TAVI is still evolving. Recent studies have explored its feasibility and safety, indicating potential benefits in minimizing vascular complications and bleeding events^[8]^. Compared to prior meta-analyses conducted by Jhand et al, our updated analysis includes additional studies and a more refined evaluation of clinical outcomes with TRA versus TFA in TAVI. The inclusion of an RCT enhances the quality of evidence, although observational studies remain predominant. Notably, our findings reinforce the reduction in major vascular complications and bleeding events with TRA, which aligns with previous studies. However, new evidence suggests a statistically significant reduction in stroke risk, which was previously inconclusive. The observed heterogeneity in certain outcomes highlights the need for further standardization in procedural techniques and patient selection criteria.

Our analysis demonstrated a significant reduction in major vascular complications with TRA, in alignment with previous studies. A meta-analysis by Jhand et al including six studies with 6373 patients found that TFA was associated with a higher incidence of major vascular complications compared to TRA (RR 0.45, 95% CI 0.32–0.63, *P* < 0.00001)^[8]^. Similarly, Garguilo et al conducted a meta-analysis involving 21 600 patients, reporting a lower risk of major vascular complications with TRA (RR 0.38, 95% CI 0.28–0.51, *P* < 0.001)^[19]^. The increased risk of vascular complications with TFA may be attributed to the use of larger sheath sizes, direct puncture of high-pressure femoral arteries, and the high prevalence of peripheral artery disease (PAD) among TAVI candidates^[20]^.PAD is present in approximately 20–30% of patients undergoing TAVI and is associated with a higher rate of vascular complications. Specifically, patients with PAD experience major vascular complications at a rate of 5.5% compared to 2.4% in those without PAD^[21]^. This increased risk is due to arterial stiffness and calcification, which can complicate vascular access and elevate the likelihood of adverse outcomes^[22]^. Repeated catheterization and insertion of large-bore sheaths in the femoral artery can lead to endothelial injury, increasing the risk of dissection, pseudoaneurysm formation, and retroperitoneal bleeding^[23]^. Our findings also demonstrated a significantly lower overall risk of vascular complications with TRA, which aligns with prior studies highlighting its advantages in reducing vascular injury (RR 0.38, 95% CI = 0.28–0.51, *P* < 0.001)^[20]^. The lower incidence of vascular complications with TRA may be attributed to the smaller sheath sizes required for transradial procedures, leading to lesser trauma to the arterial wall and a lower risk of dissection, pseudoaneurysm formation, and retroperitoneal bleeding^[24]^.

Similarly, our meta-analysis found a significant reduction in bleeding complications with TRA, a finding consistent with prior research. A systematic review and meta-analysis by Radhakrishnan et al reported lower rates of major or life-threatening bleeding in patients undergoing TAVI with secondary TRA compared to secondary TFA (RR 0.51, 95% CI 0.40–0.64, *P* < 0.00001)^[25^] [^2]^.The superficial location of the radial artery facilitates easier hemostasis, further reducing the risk of significant bleeding compared to the femoral artery^[19]^.

Our meta-analysis also demonstrated a significantly lower stroke risk with TRA, supporting previous studies that highlight the potential neuroprotective benefits of this approach^[26]^. The reduced stroke risk may be attributed to the avoidance of direct manipulation of the aortic arch and reduced embolic load during catheterization^[27]^.

Importantly, our meta-analysis demonstrated a significantly lower risk of all-cause mortality with TRA compared to TFA, reinforcing previous studies in suggesting a potential survival benefit with TRA TAVI (RR 0.71, 95% CI 0.57–0.88, *P* = 0.01)^[28]^. This reduced mortality risk may be attributed to the lower incidence of major vascular complications, bleeding events, and AKI associated with TRA^[29]^.

A significantly lower risk of AKI was also observed in patients undergoing TRA TAVI. This aligns with previous studies suggesting that TRA reduces the incidence of AKI in patients undergoing percutaneous coronary interventions (RR 0.63, 95% CI = 0.48–0.83, *P* = 0.0001)^[30]^. Moreover, our study found no significant difference in MI rates between TRA and TFA, a finding consistent with prior studies^[28]^.

We found no significant differences between TRA and TFA in terms of permanent pacemaker requirement, procedure time, contrast volume, fluoroscopy time, or length of hospital stay. These findings are consistent with previous comparative studies. For example, a meta-analysis by Jhand et al. found no significant differences in procedure time, contrast volume, or fluoroscopy time between transradial and transfemoral approaches in patients undergoing primary percutaneous coronary intervention (PCI) for ST-elevation myocardial infarction (STEMI)^[28]^.

The findings of this meta-analysis suggest that TRA could serve as a viable alternative to TFA in TAVI patients. As operators gain experience and improvements in radial-specific equipment emerge, the safety and feasibility of TRA for TAVI may further improve. Future studies should focus on long-term outcomes to evaluate whether the lower rates of vascular complications, bleeding, and AKI translate into sustained survival benefits. Additionally, patient selection criteria should be refined to identify those who would benefit most from TRA, particularly individuals with high bleeding risk, severe PAD, or previous transfemoral access complications.

Several limitations need to be considered when interpreting the results of this meta-analysis. Firstly, this meta-analysis largely comprises of observational data, which is associated with significant selection bias, therefore limiting our ability to establish causality. Although several studies employed propensity score matching to adjust for baseline differences, the retrospective nature of the included data suggests that multiple confounders could have potentially influenced the results, such as variations in baseline characteristics and a preference towards TFA due to operator experience or complexity of the case. The rarity of the outcomes prevented everal of the studies from employing robust regression models to ameliorate the impact of potential confounders. Secondly, significant heterogeneity was found in multiple outcomes. This can likely be attributed to variations in the baseline characteristics of patients (such as comorbidities and severity of stenosis) and differences in procedural characteristics (such as primary access site and method to achieve hemostasis of primary and secondary access site) across the included studies. Thirdly, long-term follow-up data was not reported as a majority of the studies focused on the immediate periprocedural phase. As such, the long term efficacy and safety of both approaches remains to be clarified. Finally, factors such as operator experience and availability of suitable equipment are likely to significantly affect outcomes, but could not be assessed.

## Conclusion

This meta-analysis of one RCT and seven cohort studies (15 462 participants) suggests that TRA in TAVI is associated with significantly lower risks of vascular complications, major vascular complications, bleeding, stroke, AKI, and all-cause mortality compared to TFA. These benefits may be attributed to smaller sheath sizes, easier hemostasis, and reduced embolic burden with TRA. Despite these advantages, TRA remains technically demanding, requiring operator expertise and specialized equipment. It may not be suit for all patients, particularly those with small or diseased radial arteries, and the feasibility of large-caliber device delivery remains a challenge.

As TRA adoption increases, challenges such as procedural learning curves, radial artery spasm, and access failure need to be addressed. Standardized training programs and advancements in radial-specific devices could improve procedural success rates. Further research needs to adjust for the limitations in the currently available data. Large-scale RCTs with robust protocols need to be undertaken to adjust for potential baseline differences which could have confounded the currently available data due to its largely retrospective nature. Future studies should evaluate long-term outcomes to determine whether the reduced risks of vascular complications, bleeding, and AKI translate into sustained survival benefits. Moreover, operator experience and availability of suitable equipment needs to be taken into account before TRA, which is likely a more technical and demanding procedure for most operators, can be recommended for widespread adoption. Additionally, cost-effectiveness analyses may help determine its broader clinical adoption. Optimizing patient selection criteria will be crucial to identifying those who would benefit most from TRA, ensuring improved safety and efficacy in TAVI procedures.

## Data Availability

The data supporting this study’s findings are available from the corresponding author upon reasonable request.

## Research Registration Unique Identifying Number (UIN)

CRD420250654859.
